# Organizational Perspectives on Access to Care Among Persons With End‐Stage Kidney Disease After Enrollment in Medicare Advantage

**DOI:** 10.1111/1475-6773.70175

**Published:** 2026-10-16

**Authors:** Denise A. Tyler, Joan F. Brazier, Ankur D. Shah, Amal N. Trivedi, Emily A. Gadbois

**Affiliations:** ^1^ Scripps Gerontology Center Miami University Oxford Ohio USA; ^2^ Department of Health Services, Policy & Practice Brown University School of Public Health Providence Rhode Island USA; ^3^ Warren Alpert Medical School of Brown University Providence Rhode Island USA; ^4^ Division of Nephrology Rhode Island Hospital Providence Rhode Island USA; ^5^ Providence VA Medical Center Providence Rhode Island USA

**Keywords:** dialysis, end‐stage kidney disease, Medicare Advantage, patient access, transplant

## Abstract

**Objective:**

To examine Medicare Advantage (MA) plan, dialysis provider organization, and kidney care management company perspectives on how MA enrollment impacts access to care among persons with end‐stage kidney disease (ESKD). The 21st Century Cures Act enabled persons with ESKD to enroll in MA, resulting in over 70% growth in enrollment among this group. However, the impact of MA enrollment on access to healthcare and services for people with ESKD remains unknown.

**Study Setting and Design:**

This was a qualitative study that included 48 semistructured interviews with 56 participants representing eight MA plans, six dialysis provider organizations, and five kidney care management companies across all US regions.

**Data Sources and Analytic Sample:**

Interviews were conducted from January 2022 to May 2024. Deductive and inductive coding methods were used and organized around Donabedian's structure‐process‐outcomes framework.

**Principle Findings:**

Perspectives diverged by organization and staff type across four themes. Regarding dialysis access, MA plan and large dialysis organization representatives reported no barriers for patients, while small dialysis providers described patients traveling farther to in‐network facilities. Concerning specialists, frontline staff reported patients were often unaware of network restrictions limiting access. Regarding transplant, MA plans noted selecting “centers of excellence,” while other participants reported these restrictions may be impeding patient access to transplants. For supplementary benefits, executives emphasized their value, while frontline staff reported benefits were often more limited than advertised.

**Conclusions:**

Participant perspectives suggest MA enrollment may differentially impact access depending on dialysis provider size and MA network restrictions. Persons with ESKD may need additional support to make informed insurance enrollment decisions.

## Highlights

- What is known on this topic
  ○ Enrollment in Medicare Advantage by people with end‐stage kidney disease increased over 70% after the 21st Century Cures Act opened enrollment to this group.
  ○ People with end‐stage kidney disease rely on frequent and complex health services, including dialysis, specialty care for multimorbidity, and kidney transplantation.
  ○ Enrollment growth in Medicare Advantage was greater among individuals who may already have healthcare access issues, including Black, Hispanic, and those dually eligible for Medicare and Medicaid.
- What this study found
  ○ Medicare Advantage plan and large dialysis organization representatives reported no dialysis access barriers for patients, while small dialysis providers described patients having to travel farther to in‐network facilities.
  ○ Frontline dialysis staff reported patients were often unaware of network restrictions limiting access to specialty care.
  ○ Medicare Advantage plan representatives noted selecting transplantation “centers of excellence,” while other participants reported these restrictions may be impeding patient access to transplants.

## 1 Introduction

The 21st Century Cures Act (hereafter the Cures Act) opened enrollment in Medicare Advantage (MA) to persons with end‐stage kidney disease (ESKD). Prior to the Cures Act, people with ESKD were restricted to traditional Medicare coverage unless they had already been enrolled in MA at the time of their diagnosis. MA differs from traditional Medicare in several important ways. MA plans are offered by commercial insurance companies who receive a per member per month payment from the federal government to provide healthcare and other supplemental benefits, such as dental and vision care, to enrollees [1]. Unlike traditional Medicare, MA plans are allowed to restrict coverage to networks of providers, require preauthorization for services, and reserve the ability to deny healthcare treatments and services entirely [2, 3, 4, 5]. Enrollees can usually obtain MA coverage for low or no premium costs, making these plans attractive to lower income individuals [6].

Since implementation of the Cures Act in January 2021, enrollment in MA by persons with ESKD has increased dramatically with an over 70% relative increase. Specifically, the proportion of Medicare beneficiaries with ESKD who were enrolled in MA increased from 25% in December 2020 to 37% in December 2021 and to 43% by December 2022 [7, 8]. In addition, enrollment growth in MA was greater among Black, Hispanic, and dually eligible beneficiaries [8], groups that may already have healthcare access issues. This growth has been accompanied by expanded marketing of MA plans to persons with ESKD, with prior research noting that the complexity of these insurance decisions may pose challenges [9].

As this transition continues, the policy's impact on how and where patients receive care is becoming increasingly consequential. People with kidney failure often rely on frequent and complex health services, including dialysis, specialty care for multimorbidity, and evaluation for transplantation [10]. Insurance design plays a critical role in whether these services are timely, coordinated, and geographically accessible. What is not known is how enrollment in MA impacts access to healthcare and other services for people with ESKD. This study sought to examine access to healthcare and services by those with ESKD enrolled in MA from the perspective of representatives of the organizations that were particularly affected by the Cures Act: MA plans, dialysis providers, and chronic kidney disease (CKD) care management companies.

## 2 Methods

This exploratory qualitative study was part of a large mixed‐methods study evaluating the effects and outcomes of the Cures Act. As part of that study, we sought the perspectives of MA plan executives, dialysis provider executives, dialysis provider frontline staff, and CKD care management company representatives with the goal of understanding implementation because these were the organizations most affected by this policy change. The results reported herein describe how these representatives thought newly available MA enrollment under the Cures Act has impacted access to healthcare and other services by persons with ESKD. Persons with ESKD were not interviewed because this was beyond the scope of the larger study. This study followed the Consolidated Criteria for Reporting Qualitative Research (COREQ) reporting guidelines [11]. The study was reviewed by the Brown University Institutional Review Board, which determined it to not be human subjects research.

### 2.1 Data Collection

We used snowball sampling techniques [12] to identify potential participants starting with a convenience sample of contacts from the full research team and colleagues. We began by recruiting executive level staff at MA plans and dialysis provider organizations. After interviewing executives at dialysis provider organizations, we also recruited facility level staff, such as social workers and insurance coordinators, for interviews. After interviews with several participants, we determined it would also be important to include executives from CKD care management companies due to the growing role of these organizations in the care of persons with ESKD. Potential participants were recruited by email.

We developed semistructured interview protocols for each type of participant, including MA plan executives, dialysis provider organization executives and facility level staff, and CKD care management company executives. The protocols included questions that were consistent across participant types and additional questions that were specific to the participant type. For example, all protocols asked, “What impact has the Cures Act had on access to care for people with ESKD?” Protocols were pilot tested with appropriate members of the larger research team and revised.

A qualitative research team composed of five women with 1–35 years of experience conducting qualitative research, including three PhD‐level faculty, one PhD candidate, and one Master's‐level research staff, conducted all interviews. The qualitative research team and interview participants did not know each other. However, one participant had participated in a previous research interview with a member of the qualitative team for another unrelated project. Interviews were conducted between January 2022 and May 2024, and two members of the research team participated in each interview on a rotating basis, with one leading the interview and the other taking notes and asking follow‐up questions as appropriate. Interviews were about 60 min. in length and conducted by telephone or video‐conference depending on the participant's preference. The audio was recorded and professionally transcribed. Participants were provided a $50 gift card in consideration of their time. Recruitment was stopped when data saturation was reached [13].

### 2.2 Data Analysis

A modified grounded theory approach using deductive and inductive methods was used to develop a preliminary coding scheme [14], meaning that some coding categories were developed a priori based on our interview questions and others were developed de novo in response to concepts identified during coding [15, 16]. Each member of the qualitative team coded the first few transcripts line by line [14] using the preliminary coding scheme, and the team met to discuss the utility of the coding scheme, after which the coding scheme was reorganized based on Donabedian's structure–process–outcomes framework [17], so that these constructs would be consistently captured across participants. Codes continued to be added, deleted, and refined until a final coding scheme was agreed upon.

Every transcript was then coded by four members of the qualitative team. The team met to review coding and come to final decisions about the coding of all qualitative data. During these meetings, qualitative themes were also discussed, and an audit trail of all team decisions was kept to ensure rigor [18]. NVivo, version 12 Plus (QSR International) was used for management of the coded data.

## 3 Results

We conducted 48 interviews with 56 participants from eight MA plans, six dialysis provider organizations, and five CKD care management companies. Most interviews included one participant; five interviews included more than one participant. Representatives from national and regional MA plans, dialysis providers, and CKD care management companies were included representing all regions of the United States. See Table 1 for details about these organizations and participants. MA plan representatives included executives, such as Chief Medical Officers and Vice‐Presidents or Directors of Government Relations, Care Management, and Clinical Operations. Dialysis provider representatives included Chief Medical Officers, Chief Financial Officers, Directors of Communication, and in the case of small providers, owners. Dialysis facility staff included social workers, insurance/billing coordinators, and nurse care managers. CKD care management company representatives were primarily Chief Medical Officers. Some organizations used unique titles that could be used to identify participating organizations. Therefore, we report only generic titles in Table 1.

**TABLE 1:** Organization characteristics and participant titles/experience.

MA plan	National/regional	Region	Size/enrollment	Participant titles	Years in role
MA 1	National	National	1–5 million	Chief Medical Officers VP/Directors Government Relations VP/Directors of Care Management VP/Directors of Clinical Operations	1–5 years
MA 2	Regional	Midwest	1–5 million		
MA 3	National	National	> 5 million		
MA 4	Regional	West	< 1 million		
MA 5	Regional	Southeast	< 1 million		
MA 6	Regional	West	> 5 million		
MA 7	Regional	Northeast	< 1 million		
MA 8	Regional	Midwest	< 1 million		

CKD care management	National/regional		Size/enrollment	Participant titles	Years in role
CKD 1	National	National: 50 states	> 100,000	Regional/Chief Medical Officer Chief Clinical Officer	< 1–5 years
CKD 2	National	National: 50 states	> 100,000		
CKD 3	Regional	National: < 50 states	> 100,000		
CKD 4	Regional	National: < 50 states	> 100,000		
CKD 5	Regional	National: < 50 states	< 100,000		

Dialysis provider	National/regional	Region	Size	Participant titles	Years in role
DPO 1	National	National	> 100,000	*Leadership* Chief Medical Officers/Financial Officers VP/Directors of Insurance Coordination Directors of Communication Owner/operator	2–23 years
DPO 2	National	National	> 100,000		
DPO 3	Regional	Northeast	< 100,000		
DPO 4	Regional	West	< 100,000		
DPO 6	Regional	Southeast	< 100,000		
DPO 7	Regional	Midwest	< 100,000		
				*Staff* Social Workers Insurance/Billing Coordinators/Specialists Patient Accounts Managers Nurse Care Managers	

Abbreviations: CKD, chronic kidney disease; DPO, dialysis provider organization; MA, Medicare Advantage.

Qualitative analysis identified four main themes: access to dialysis, access to other providers, access to transplant centers, and access to supplementary benefits, such as transportation, dental, and vision benefits. Perspectives diverged by organization and staff type across these themes. Illustrative quotes are provided in the text and in Table 2.

**TABLE 2:** Quotations by theme and subtheme.

Themes and subthemes	Quotation	Participant
1. Access to dialysis		
MA plans and large DPOs reported that the policy change did not create new barriers to access	I think access has probably been increased because most of these plans do have very good networks overall. So hospitals and providers are plentiful and available	DPO1.P2
	We were having to have contracts with so many other little facilities across the state where if we could have just signed with those two big ones, it would've met our network requirements then. Now, we have both. We took the time to do all those little network ones and give members those options. And then now, we have the two big ones as well	MA2
	I'm not aware of any issues with access	MA5
	Because they're picking a new plan, that affects their rights to request continuity of care and yet dialysis is generally time sensitive. So you can't go too long without receiving a treatment, without having harm. And providers like the dialysis centers are not going to want to provide the care without an authorization because then they can be denied for no prior authorization and get no payment. But to my recollection, that actually went pretty smoothly. I don't recall any patients having care coordination issues, even though theoretically they could have	MA6.I2
Small DPOs reported barriers to access	A decrease in reimbursement during a time of some of the highest inflation I've seen in my life. We've seen centers closing, especially rural ones. And so, access to care is becoming more difficult. We're seeing choice, patient choice eroding and more barriers to care emerging	DPO6.P1
	I feel like some of the plans have erected barriers in terms of access to facilities closest to the patient's home and forcing them to use units that are farther away because they're out of network and they're considered out of network. And so I don't think the plan should have any say in where a patient receives dialysis or what modality …	DPO8.FLS2
Dialysis providers find out patients switched only after payment denied	So the pharmacy would call and say, there's something wrong with the patient's insurance. Then I in turn will get in contact with the financial coordinator and say, what's going on with the patient's insurance? She'll look into it. She has a system where she can see everything and she'll tell me whether it lapsed or whether they didn't make a payment or whatever, they switched	DPO1.FLS4
	And it gets nasty when a patient switches back and forth and back and forth, excuse me, just because of the hospital and all the providers and making sure that everybody knows you've changed your plan around	DPO1.FLS11
	Because from my end, so I'm billing the insurance that I think you have, I'm getting the denial. Now I've got to backtrack and see, where is this patient at now? And so we bill the new insurance that they're supposed to have, and if for some reason that comes back as we're not in network, well, that's when I have to turn around and get the social worker involved and say, “Apparently the patient picked a plan that we're not contracted with, so we're not going to get paid. So either they have to choose another facility, or they have to choose another plan”	DPO6.FLS2
	So the patients switch because they got a gift card or something. And when they let our financial counselors know, by the time they let us know, or we got to get authorization. And then we find out we're out of network, they're only allowing them to stay here for 90 days for the authorization	DPO7.FLS1
Dialysis provider insurance coordinators have to fix the problem	I had a patient who did this and his insurance coverage was changed in the middle of his transplant referral stage. And, unfortunately, the center did not accept the insurance he was enrolled in after this. So we had to kind of put that on hold and go back and try and fix it	DPO1.FLS3
	And I actually have a case like this, that's why I'm saying it, is where we weren't aware that the patient switched. However, this patient has already been established in dialysis. And that's my hope, that instead of taking it from the day they receive my request for an authorization, that they backdate it to the previous month, because there again, they were already on dialysis. It's just the patient changed to … I mean, it's just a matter of continuity of care. We're not going to … And like I said, a lot of times patients do not let us know necessarily. Very rarely do we have those who come in and say, “I'm going to have a new insurance that begins next month.” And I mean, very rarely. It's always after the fact	DPO6.FLS2
	I'll contact the nephrologist or whatever doctor it is that's not in‐network, try and get something worked out. The patient wasn't aware that you were in this, and try and get the patient back enrolled into the previous plan that he had before he went into this [MA] plan where it's nothing being covered	DPO7.FLS2
Organizations report trying to improve access to home modalities	They're saying, “Hey, show us, are you moving the needle? Are you getting more people preemptively transplanted? Are more people moving toward home?” But what they're really looking at is the total cost of care bottom line. What we know though is that patients who are preemptively transplanted, patients who are dialyzing at home, their total cost of care and quality care is better. That's what they also look at	CKDM3
	… through that additional care coordination we did see an impact in helping more patients to choose to treat on a home modality. I think that was less of a reflection of differential education, and I think more of an impact coming from the regional payers helping to remove barriers	DPO2.P1
	We encourage home dialysis. [DPO4] is we have a very high percentage of patients on home dialysis in comparison to the national average, but probably we could even do better. So we are strong encouragers of home, especially with the pandemic, we want to emphasize to patients, you don't have to sit in an environment with a lot of people that are relatively close by… Nonetheless, patients can do dialysis at home if they like, and we encourage that growth. And so MA I'm sure likes our dialysis organization because we have a lot of home …	DPO4.P1
	We're all onboard! We think it's great. Obviously I'm not a clinician. We have a lot of smart doctors sort of working on that. Even on our website, I think we've got a lot of data and information and just resources about the benefits of home dialysis and sort of the quality and safety aspects to it, and the convenience, obviously, for those patients where it works, because it may not work for everyone because not everyone may want to actually do home dialysis, so it's going to be patient specific	MA1
	As far as the in‐home dialysis, working with the [State] Renal Association, they provided a lot of great opportunities for us to partner with some of the nephrology centers. One nephrologist in particular, that we'll be meeting with soon, half of all of his new patients go directly to in‐home dialysis, so just really seeing what he's done and gained from his wins. And that way, we can develop that into our program too. And then that letter that I told you that was going out to all of the dialysis facilities, just making them aware. And we have a letter that goes out to all of our nephrologists as well that makes them aware that [MA2] supports home dialysis, and then asking them what we can do to help support that in their decision making	MA2

	I mean our best thing that the MA plan can do to encourage home dialysis is to partner with these early stage CKD companies that are focused on starting it the right way, because there's going to be people that progress to dialysis no matter what, and that's fine, but if you want someone to get home dialysis, this chance to talk to them 6 months before they need dialysis and start training them, training their caregiver, assessing their home, to make sure they have the space to do it and getting them set up. And so you want them prepared to do home dialysis well in advance of when they actually need it. And so the MA space our ideas in incentivized contracting, that's the way we influence it	MA3
	So our strategy with working with these partners was to make sure that the partners aligned with our vision on improving adoption of home dialysis. That was formal. While that we don't have a policy that forces anyone to do home dialysis or anything like that, but making sure the education, the support system, if someone chose home dialysis, was sort of there	MA3.I2
	I don't know that they're actively in [State] yet, but we do look at, as I'm sure all the players do is this idea of at home dialysis is really that there could be some health benefits there. It's easier for the member to not have to go to the center. There could be some potential cost savings	MA4
	Nothing at the moment other than working with members to try to encourage them to move to that [home modality]. We have not provided any financial incentives. I question whether there's a potential moral hazard in doing that. I think we try to make it available, encourage, educate, but not at this point incentivize	MA5
I'm sure that it's addressed through some of the benefit design, but when we do these contracts, what we're looking for is obviously improvement in those metrics, whether it's an in‐home dialysis start or an in‐center dialysis start or just an appropriate start generally	MA8
2. Access to other providers		
MA plans have limited provider networks	And a lot of that had to do with, there are other providers not being in network, cardiologists and things like that. Not necessarily the nephrologist or us. Or, the transportation provider might not have been networked with the plan and they required the transportation to get to their dialysis treatments	DPO1.P2
	I've had some that did it because maybe their provider wasn't in network with their new plan, so they had to switch to another one. They found out that their provider doesn't accept [one MA plan] or [another MA plan] whatever. Some did want to switch back. They switched back to just traditional Medicare because they were able just to go back to what was before, and they just didn't like that they had to switch doctors …	DPO1.FLS2
	I would just echo the point about having a sufficient provider network. I think that's potentially one of the big tradeoffs in moving from traditional Medicare is you have to sure that you can continue to see the nephrologists and dialysis providers and other specialists of choice	DPO2.P2
	We've had difficulties with a patient getting them in to see an interventional radiologist to get access work done in a timely manner. So, what could have been something easy turned into something a lot harder	DPO6.P1
	I think negative benefits would be potential barriers to accessing preferred units or preferred providers	DPO8.FLS2
Patients were frequently unaware of MA network restrictions	Some have lost their providers, they get on a plan and their providers aren't covered and they disenroll so they can get back to their providers… patients are just poorly informed about it. And I think most patients don't like that because they're used to having very free access to any provider they choose under traditional Medicare	DPO1.FLS7
	Yeah, it's more their other doctors so like, “I've been seeing the same cardiologist for this many years and now I can't see them,” kind of thing	DPO1.FLS10
	And then occasionally, patients would find their nephrologist is not in network with the plan they switched into. And that really created some angst	DPO1.P1
	And I'll say most of the feedback that we got was related to contracting where they said, “Well I believe that I was contracted with X, Y, and Z. Turned out my heart doctor wasn't contracted.” So I'm their more than … I had to weigh out what was more important to me.” I think for others I would say contracting's the number one issue. Because again, we're only one of their providers. And so if they found that some, one of their other physicians is not covered, that could be a problem for them	DPO3.FLS3
	I was just talking to the billing person this morning about a patient that switched to a Medicare Advantage plan, and we're not covered. We're still treating him, but he had to switch his family doctor to a different doctor. He's had issues with his medications, and he's just one example of someone that's kind of had to jump through a bunch of hoops because they switched to a Medicare Advantage plan that didn't include all of the people that care for them	DPO6.FLS1
	Well, a lot of them, sometime they'll switch for that extra $20 and then they'll find out some of their doctors are not in network. And then they're, “Oh, my doctor ain't in network, [Participant]. I'm getting a bill.” “Why did you switch your insurance?” “Well, they was giving me an extra so much.” And then they have to turn around and go back into the old plan. Like I said, it's just back and forth with them sometimes. Go back into the old plan because one of their doctors is not in‐network and now they're receiving a bill from the doctor that's not in‐network	DPO7.FLS2
3. Access to transplant centers		
MA plan networks restrict access to transplant centers	Or not four or five but ever since dialysis patients could sign on to MA plans, we were told like, “Tell your patients how great Medicare Advantage is, because the reimbursement's a little bit more” for the dialysis unit. Not for the physician but for the dialysis unit, they got a little bit more. And they didn't really touch on the fact that some of your transplant‐eligible patients may find that their preferred‐transplant provider is now out of network, for example	CKDM4
	So some MA plans only contract with one place in a whole state or in a region, which makes it very difficult, get someone transplant. So they need to really improve their networking adequacy and access to help these patients in my opinion … For example, we have a patient that we're following right now in [state] who's an MA patient and there's no transplant center in [state], that's in network with this provider. So the patient had to go to [another state] for a transplant evaluation, which is a huge hurdle. So they need to do better and either negotiate rates and providing access to doing single patient agreements for those members, that transplantation is an option for	CKDM5
	Because if that transplant center doesn't take that insurance plan, then of course, no, it won't be offered to you. In a sense, it's just like any issue you would have with a insurance provider, if it's in network, out of network type of situation	DPO1.FLS5

	With transplant, it does in terms of you're in a network. So yes, there's only certain transplant centers that are within that network, so we do have to ensure that we only go to those, and that's not necessarily giving you … With the straight Medicare, you just get the choice of whichever transplant center you would like. So it does limit you in that way	DPO1.FLS10
	Finding out that your transplant center was no longer willing to talk to you because you had changed to some plan that they weren't welcome in	DPO1.P1
	… I know folks that have had to switch transplant centers because of their insurance	DPO6.FLS1
	I know we have in the past run into patients who have had to change, because whatever coverage they had didn't provide transplant services for ESRD patients. So if they were looking to get on the transplant list, they would have to change their insurance	DPO6.FLS2
And the transplant centers will tell them right upfront, “don't change your insurance, whatever you do don't.” It's not uncommon for us to have one patient submit five applications and see which one sticks, which one's going to accept the first. And then, the plans seem to be getting involved in saying, “Well, we work with that one, we don't work with this one”	DPO6.FLS3
MA plans restrict access to “centers of excellence”	The truth is though, that if a patient is in Medicare Advantage, they will likely have a better, more robust set of benefits to get them, for example, to a transplant. Centers of Excellence that are in the provider's network, providers who work with their physician partners on extra care management steps to get patients into those Centers of Excellence and stuff like that	CKDM1
	Yeah, I mean for transplant, there's centers of excellence that might be preferred to doctor or so forth …	CKDM2
	It doesn't seem to be as big of an issue in that regard, but I certainly have seen it to where they limited, they only work with … center of excellence. You must use that. It eliminates other options the patient might have, for whatever reason	DPO1.FLS7
	And in almost every MA Plan, the plans will have what they call “centers of excellence,” which is code for, we have a relationship with this particular transplant center. Typically, those “centers of excellence” are actually picked because they do have good outcomes, which means they also have lower costs. And that's usually okay. Where we see people running into problems is if they're already enlisted somewhere else and the plan that they were talked into signing does not consider the facility that they're used to going to, and already have a relationship with, as a “center of excellence.” And that creates some real problems for patients	DPO1.P1
	So how that works out here, even for us of course, we have contracts with centers of excellence and then we have an overarching contract that is supported by a large entity across the country that contracts with transplantation facilities for all sorts of things, not just kidney transplant … We felt most comfortable with [Hospital 1] and their transplant facility, and they work with us really well. And so that's our center of excellence	MA6
4. Access to supplementary benefits		
Availability of these benefits could be very beneficial to this population	I think the ability to provide other types of services, especially around behavioral health and social determinants and things like that, is a pretty big win. And so the way I see it is that any value‐based arrangement will be good, will be as good as the opportunity and the ability to provide all these ancillary services that patients need to be able to deliver them appropriately	CKDM2
	I think my thoughts here are MA plans can really help improve social and structural inequalities in healthcare. For them, it's a business imperative. I think it's an ethical imperative on my part, but it's also a business imperative for them. Whereas in fee for service there is not, because again, the system was essentially created based on relative value, therefore you see a patient, you get a check, you move and do the next thing. But I think for MA, for sure there's a business imperative to address this, which is why a lot of MA plans have moved toward these interesting benefit plan design structures to really get at and address social and structural issues	CKDM3
	It can be a wonderful opportunity for patients that don't have the ability to get a secondary, because it does cover the Medicare and the supplement all rolled into one. It also has a low premium. So if it's a state that doesn't have the ability to have a secondary and there are states out there with no secondary ability, this is a wonderful opportunity	DPO1.FLS11
	Having a choice of transportation companies in their network is another big one because these patients, some of them need to have transportation to get to dialysis treatments. And, in my mind, it is a benefit also to the plan to have that available. Because if somebody doesn't go to treatment, they're going to have a bad outcome, which is going to cost them more money in the long run	DPO1.P2
	I think what Medicare Advantage did was it gave those patients, if they live in a certain area, the opportunity to have more robust benefits than just Medicare only	DPO1.P3
	What our patients have really found attractive are benefits like dental coverage, for many of our patients who are receiving dental coverage literally for the first time ever. I don't have an exact data point, but we hear a lot of anecdotes about patients who are able to get dental coverage, which is particularly important for patients who are seeking a kidney transplant for infection control reasons post‐transplant	DPO2.P1
	And then more specific to our patient population, there's some of the more social determinant of health type benefits that have proven to be interesting to many patients. Things like transportation coverage to improve patient's ability to access not only their dialysis provider, but other healthcare. And meal benefits or healthy meal counseling services that some of our patients receive are a few other examples	DPO2.P3
	So with regard to the transplantation services issue and sometimes when we've asked that we've heard back that MA sometimes has better dental benefits than traditional Medicare does. And so for those reasons, MA might be helpful in getting people eligible for transplantation because those dental services need to be handled prior to transplantation	DPO3.FLS1
	So I think positive things are the access to benefits that traditional Medicare doesn't necessarily cover. I think that's a great positive	DPO8.FLS2
Promise of these benefits entices people to switch to MA	That they were trying to get the dental, the vision, and the extra food assistance, trying to get additional resources	CKDM5
	Most of them, they do change due to supplemental benefit, transportation benefits, vision benefits, dental. If they do want to change, that's one of the biggest reasons they want to change. The scam‐wise, I just tell them to be really careful because it's really hard to know over the phone, but I usually tell them if you get a letter from something that you feel is suspicious, or you want me to look over it, I usually tell them to bring it in the clinic and I kind of look over it with them before	DPO1.FLS6
Supplementary benefits were often not as promised	Some of our patients need cataract surgery, need glasses, they need drops. You know. It's just ongoing stuff that they need, and $100 extra doesn't really go a long way. Dental work, $100 doesn't really help a whole lot	CKDM5
	And under this plan, he had to utilize their transportation. So the process changed. He had to set it up in advance. They had to do it weekly. Then when they would do it and they would set everything up and they would get the confirmation, he and his wife would call me probably two out of three days every week saying, “My ride didn't show up”	DPO1.FLS2
	I've seen numerous examples of a patient getting connected to a plan that's specific for the [City 2] market, and all of our patients and our community is connected to the [City 1] market. So they're getting connected to plans where they don't even … they've never gone to [City 2] for healthcare services. They've always gone to [City 1]. They're getting connected to plans that just don't work or fit for even the basic of their needs	DPO1.FLS7
	So the plan may state that they have vision and dental and transportation, but when you actually go and look at what's available in your county, those are not available	DPO1.FLS11
	But no one else has been able to utilize transportation services. It seems like it's something that's advertised, but then I don't know if it's because we're in a small rural area, it's just not something that people can take advantage of	DPO6.FLS1
	So I think that they get taken in. Or some of the other dental, but yet nobody's taking the insurance	DPO8.FLS1

### 3.1 Access to Dialysis

Analysis revealed that representatives from MA plans and executives from the large dialysis providers were more likely to report that the Cures Act policy change and enrollment in MA did not create barriers to dialysis for ESKD patients. One MA plan representative said, for example, “I'm not aware of any issues with access” (MA5), while an executive from a large dialysis provider said, “I think access has probably been increased because most of these plans do have very good networks overall” (DPO1.P2). Conversely, small dialysis provider representatives were more likely to report that “access to care is becoming more difficult” (DPO6.P1). As one frontline staff member reported, “I feel like some of the plans have erected barriers in terms of access to facilities closest to the patient's home and forcing them to use units that are farther away …*”* (DPO8.FLS2).

Regardless of dialysis provider size, frontline staff at dialysis providers reported that they sometimes discovered that patients had enrolled in an MA plan after payment for dialysis services was denied. They described the steps they needed to take to correct this issue. As one explained, “… so I'm billing the insurance that I think you have, I'm getting the denial. Now I've got to backtrack and see, where is this patient at now?” (DPO6.FLS2). Dialysis provider insurance coordinators described the work they then needed to do to fix the problem:

I'll contact the nephrologist or whatever doctor it is that's not in‐network, try and get something worked out. The patient wasn't aware that you were in this and try and get the patient back enrolled into the previous plan that he had before he went into this [MA] plan where it's nothing being covered. (DPO7.FLS2)

Across organizations, executive level participants discussed increased access to home modalities. MA plan representatives reported that they are “working with members to try to encourage them to move to [home dialysis]” (MA5). They did this by providing information directly to patients. For example, “on our website, I think we've got a lot of data and information and just resources about the benefits of home dialysis*”* (MA1). Other MA plans directed communications to nephrologists. As a plan representative explained:

… we have a letter that goes out to all of our nephrologists as well that makes them aware that [MA2] supports home dialysis, and then asking them what we can do to help support that in their decision making. (MA2)

Performance in contracts between CKD care management companies and MA plans was often judged, in part, on increasing patient use of home modalities. MA plan representatives reported expecting the care management companies to ensure patients were “prepared to do home dialysis well in advance of when they actually need it*”* (MA3). Care management company representatives said that this was because “patients who are dialyzing at home, their total cost of care and quality of care is better” (CKDM3).

### 3.2 Access to Other Providers

Participants from dialysis providers of all sizes discussed access to other healthcare providers, especially specialists, and noted that MA plans have limited networks of providers which may impact patient access. For example, a representative from a large dialysis provider said that “there are other providers not being in network, cardiologists and things like that. Not necessarily the nephrologist or us*”* (DPO1.P2). A representative from a small dialysis provider noted that “negative benefits [of MA enrollment] would be potential barriers to accessing … preferred providers” (DPO8.FLS2). A representative from another small dialysis provider said, “we've had difficulties with a patient getting them in to see an interventional radiologist to get access work done in a timely manner. So, what could have been something easy turned into something a lot harder” (DPO6.P1). Another large dialysis provider representative summed up the issue by saying:

I think that's potentially one of the big tradeoffs in moving from traditional Medicare is you have to ensure that you can continue to see the nephrologists and dialysis providers and other specialists of choice. (DPO2.P2)

According to dialysis provider front line staff, patients were frequently unaware of MA networks restricting whether or not their preferred healthcare providers were in‐network. As one front line staff participant reported, “patients are just poorly informed about it*”* (DPO1.FLS7). In particular, the differences between network restrictions required by MA versus the unlimited choice of providers under traditional Medicare seemed to be poorly communicated and understood. One frontline staff participant explained:

We've heard patients say to each other and to us, “Well, I'm not sure why my plan is not covering my cardiologist. They told me when I was applying for the plan that it would cover my cardiology care.” And I would, at that point, explain to them, it does most likely cover cardiology, but they needed to check to see if your cardiologist was in network with the plan. (DPO3.FLS3)

### 3.3 Access to Transplant Centers

There were also differences across organizational types in how participants described access to transplant services. MA plans said “we have contracts with centers of excellence” (MA6) and these were selected based on patient outcomes. A CKD care management company representative noted similarly that these contracts result in “extra care management steps to get patients into those centers of excellence” (CKDM1). However, a representative of a large dialysis provider couched this differently:

And in almost every MA plan, the plans will have what they call ‘centers of excellence,’ which is code for, we have a relationship with this particular transplant center. Typically, those ‘centers of excellence’ are actually picked because they do have good outcomes, which means they also have lower costs. (DPO1.P1)

Dialysis provider frontline staff also reported that “there's only certain transplant centers that are within that [MA plan] network” (DPO1.FLS10), and “there are certain Medicare Advantage plans that some transplant centers just do not contract with” (DPO1.FLS3). They were more negative about these restrictions than MA plan representatives, reporting that these restrictions can cause issues with access to transplant for some ESKD patients “because if that transplant center doesn't take that insurance plan, then of course, no, it won't be offered to you” (DPO1.FLS5). Similarly, if the patient was already on a transplant wait list and then enrolled in an MA plan, they may find “your transplant center was no longer willing to talk to you …” (DPO1.P1). In response, some participants reported that transplant centers warn patients “don't change your insurance, whatever you do …” (DPO6.FLS3).

### 3.4 Access to Supplementary Benefits

Finally, participants discussed patient access to supplementary benefits, such as dental, vision, and transportation. While some participants were more positive about these benefits, most frontline dialysis provider staff reported that patients had difficulty accessing them. Supplementary benefits were often cited as a key reason for enrollment in MA since traditional Medicare does not provide these benefits. Participants noted that for some patients supplemental benefits are “one of the biggest reasons they want to change” (DPO1.FLS6) to MA. Some participants, especially executives, reported that the availability of supplementary benefits could be very beneficial for those with ESKD. For example, a representative for a large dialysis provider said that “transportation coverage… improve[s] patients' ability to access not only their dialysis provider, but other healthcare” (DPO2.P3).

Other participants, particularly frontline dialysis staff, reported supplementary benefits were often not as promised and that patients were **“**getting connected to plans that just don't work or fit for even the basic of their needs” (DPO1.FLS7). Several participants provided examples of benefits amounts being very low and that for “dental work, $100 doesn't really help a whole lot” (CKDM5.P1). Others reported that benefit amounts may be fine, “but yet nobody's taking the insurance” (DPO8.FLS1).

Several dialysis frontline staff lamented that transportation benefits were “something that's advertised, but then… it's just not something that people can take advantage of” (DPO6.FLS1). Summing up, another frontline staff person said, “the plan may state that they have vision and dental and transportation, but when you actually go and look at what's available in your county, those are not available” (DPO1.FLS11).

## 4 Discussion

Our study sought to determine, from the perspective of the organizations most involved, how access to healthcare and other services has been impacted by ESKD patient enrollment in MA after the Cures Act. Interview participants reported that MA enrollment is reshaping access to care for people with kidney failure, but perspectives on this transformation varied by organizational type and staff role. MA plan representatives and executives from large dialysis organizations generally reported few access concerns, while representatives from small dialysis providers and frontline staff more frequently described barriers impacting patients. These divergent perspectives were evident across all domains we examined: access to dialysis, other healthcare providers, transplant centers, and supplementary benefits.

MA plan representatives and executives from the large dialysis providers reported no issues with access to dialysis providers, but representatives of small dialysis providers expressed that patients sometimes had to travel farther to access in‐network facilities. Perspectives about access to dialysis may have been more negative among small dialysis providers due to their experiences contracting with MA plans. Previous research has shown that after the Cures Act, the two largest dialysis providers had 33–40 percentage points higher participation in MA networks compared to independent, not‐for‐profit facilities [19]. The lower MA participation rates among small independent dialysis providers, therefore, may indeed result in reduced access for some MA enrolled patients as these patients may need to travel farther to receive dialysis at an in‐network facility. Upon switching to MA, ESKD patients utilizing these small independent providers would be more likely to lose access to their preferred facility.

Executives across organizations reported that MA plans are actively working to expand home dialysis access through strategies such as patient‐facing educational materials, direct outreach to nephrologists, and performance metrics embedded in contracts with CKD care management companies. This emphasis aligns with broader policy goals, as the United States has historically lagged behind other developed nations in home dialysis utilization [20], and expanding access could benefit ESKD patients given evidence that home modalities are associated with increased patient autonomy [21, 22], quality of life [23], and reduced transportation burden [24]. However, our qualitative findings cannot determine whether these efforts are translating into actual increases in home dialysis uptake; participants described intentions and strategies, but effectiveness remains unknown. Quantitative research is needed to evaluate whether MA‐enrolled ESKD patients are more likely to utilize home dialysis compared to those in traditional Medicare and whether MA plans' stated commitment is producing meaningful changes in care delivery.

In terms of access to other healthcare providers, frontline dialysis staff noted that ESKD patients often had limited understanding of the network restrictions allowed under MA. These participants reported that patients selected MA plans without first determining if their preferred providers, and especially specialists (e.g., cardiologists), were included in the MA plan's network. This finding is particularly notable given the breadth of specialty care required by persons with ESKD. Beyond nephrologists and dialysis providers, these patients often need ongoing relationships with vascular or peritoneal access surgeons, interventional radiologists, transplant teams, cardiologists, endocrinologists, and others to manage their complex medical needs. The potential for network restrictions to disrupt any one of these relationships, often without the patient's awareness at enrollment, underscores the stakes of insurance decisions for this population. Other research has found that many people struggle to make insurance decisions that are in their best interests [25], that Medicare beneficiary understanding of their insurance choices is lacking, and that vulnerable beneficiaries need more assistance and information [26].

Many participants also noted MA plan use of transplant “centers of excellence.” MA plan representatives reported that these providers were included in their networks due to their superior outcomes. However, other participants suggested that a key outcome was cost. While directing patients to high‐performing transplant centers could benefit outcomes, network restrictions that limit transplant center choice raise important concerns. Given the known increase in survival for ESKD patients who received transplants [27, 28], additional research is needed to determine how enrollment in MA is affecting patient access to transplants.

Our most disparate responses were related to access to supplementary benefits, such as dental and vision coverage, and transportation services. Many participants, especially those at the executive level, noted that access to supplementary benefits has the potential of being very beneficial to ESKD patients. ESKD patients, for example, often need dental work before kidney transplantation [29] and require assistance with transportation to dialysis [30]. In contrast, frontline dialysis staff, especially, reported that while supplementary benefits were advertised by MA plans, they were often limited in practice. In some cases, coverage amounts were so low it was essentially useless, or network restrictions meant no providers in the area were providing the benefit. This is consistent with previous research indicating that ESKD patients are often enticed to switch to MA plans by these promised services [9].

Our study has some limitations. We used snowball sampling techniques to recruit participants for interviews, and different recruitment techniques may have resulted in different participants who may have reported different information. However, our findings were consistent across organizations that varied in terms of geography, size, and organizational type. Also, participants may have considered some of the information we were asking about to be proprietary and withheld some details that could have affected our conclusions. In addition, many of the results shared, especially by organizational leaders and executives, may have been aspirational and not actually realized. For example, there seems to be a clear intention to grow use of home modalities, but additional research is needed to determine if these outcomes have been achieved. Finally, our large mixed‐methods study focused on the impact of the Cures Act on the organizations most affected by this policy change—MA plans, dialysis providers, and CKD care management companies—and it was their perspectives about patient access reported here. Future research should include the perspectives of persons with ESKD, which was outside the scope of this project.

In conclusion, enrollment in MA by persons with ESKD after the Cures Act may be impacting their access to healthcare and other services. Beneficiaries considering enrolling in MA plans should be aware that network restrictions can limit access to preferred dialysis and other providers. Persons with ESKD make up a particularly vulnerable segment of the Medicare beneficiary population and may need additional support and information about the implications of enrolling in MA to make informed choices that safeguard their access and outcomes.

## Funding

This work was supported by National Institute of Diabetes and Digestive and Kidney Diseases (R01DK129388).

## Conflicts of Interest

The authors declare no conflicts of interest.

## Data Availability

The data that support the findings of this study are available from the corresponding author upon reasonable request.
